# Stimulating ambulance specialist nurse students’ ethical reflections by high-fidelity simulation

**DOI:** 10.1177/09697330241291162

**Published:** 2024-10-15

**Authors:** Jonas Wihlborg, Ulf Andersson, Anders Sterner, Lars Sandman, Anna Kängström, Gabriella N. Boysen

**Affiliations:** Dalarna University; 1802 University of Borås; 4566 Linköping University; 1802 University of Borås

**Keywords:** Ambulance care, ethical reasoning, ethical reflection, simulation, specialist education, nursing

## Abstract

**Introduction:** Ethical competence in professional practice can be considered essential among nurses and nurses in ambulance care encounter ethical dilemmas frequently. To enhance ethical competence among students in the ambulance specialist nursing program, high-fidelity simulation scenarios including ethical dilemmas were introduced as a learning activity. **Research aim:** The research aim was to investigate the usefulness of high-fidelity simulation in ambulance specialist nurse education to teach ethical reasoning when caring for children. **Research design:** This study was conducted as a qualitative interview study, complemented with observations and using field notes and qualitative interviews for data collection. Data was analysed using deductive qualitative content analysis based on a care ethical model. **Participants and research context:** Participants (*n* = 35) were recruited from an ambulance nurse educational program at a Swedish university. Data was collected after the students took part in two high-fidelity simulations involving children in an ambulance care setting. **Ethical considerations:** The study has been vetted and approved by the ethical council at the University of Borås, Sweden. The study follows the Helsinki Declaration’s advice on ethical principles. **Results:** The results showed that most of the students expressed some form of ethical reasoning during the simulation sessions, which were elaborated and reflected upon during the debriefing part of the sessions. The simulation design seemed to have a great impact on the outcome of the student’s ethical reasoning, where increased immersion led to deeper emotional engagement among the students which increased awareness of their personal preconceptions. **Conclusions:** This study aimed to investigate whether high-fidelity simulations could be useful to stimulate ethical reflections and contribute to increased ethical competence among students. In conclusion, a well-designed high-fidelity simulation can be useful as an educational tool to learn and enhance ethical competence among specialist ambulance nursing students.

## Introduction

Ethical competence in professional practice can be considered essential among nurses in general. Following Rest and Narvaez,^1^ ethical competence includes moral sensitivity to identify ethical issues, moral judgement to arrive at conclusions about what to do, moral motivation to act on these judgements and moral character to be persistent in acting ethically. Nurses in ambulance care encounter ethical dilemmas frequently and demands on their ethical competence are comprehensive.^2^ They are expected to act based on the ICN code of ethics for nurses,^3^ which regulates ethical values, responsibilities and professional obligations as a nurse. In Sweden, ambulances are staffed by at least one registered nurse (RN) or specialist nurse (SN). There is no national requirement that RNs in ambulance care must be qualified as SNs, although a second-cycle specialist education for ambulance nurses is available where the students obtain a master’s degree in caring science.^4,5^ National requirements that regulate the minimum education and training for ethically demanding situations in the profession are lacking and the SN education program addresses this by introducing valuable skills training necessary for ambulance nurses to enhance their ethical competence.^6^ Within the ambulance nursing program, a flexible learning approach is commonly used which includes different pedagogical models and didactics. Examples of these are 1) Flipped Classroom,^7^ where students independently acquire theoretical knowledge through films, online courses and reading course literature, 2) Case methodology^8,9^ is used in the learning process in various forms, when students work with patient cases and share the knowledge they have acquired, for example, at seminars or simulations, and 3) Experiential learning as a model to bridge theory and practice, including for example, clinical practice and simulation at the university. Since the overall view of knowledge is fundamentally formative within the education program and the teaching activities are characterized by active and flexible learning, the assumption is that active learning, including simulation, could be adequate as a successful learning strategy.^10^ Experiences of teaching in the ambulance nurse educational program have nevertheless contributed to identifying difficulties in developing learning activities to enhance students’ ethical competence.^11^ Caring for children in the ambulance service is relatively rare.^12^ Thus, it is valuable that students are confidently offered simulation involving the vulnerable patient group that children constitute.^13^ In this study, it was assumed that if the students were exposed, two and two at a time, to simulated situations where they were forced to make ethical decisions and then reflect on them together with facilitators and a few students who observed the scenario (*n* = 1-2), then their ethical reflections would be elaborated and considered.

### Simulation

High-fidelity simulation means the degree to which the simulated environment (e.g. manikin, room, tools, equipment, moulage, and sensory props) replicates the reality and appearance of the real environment.^14^ Simulation-based education has emerged as a valuable tool in ambulance nurse education, offering an opportunity for students to engage in realistic scenarios and actively participate in hands-on experiences.^15^ By simulating real-life emergency situations, ambulance nurse education simulation allows students to develop and enhance their clinical skills, critical thinking abilities, and decision-making processes. This form of education is particularly valuable in the field of ambulance care, where providers often face chaotic and information-poor environments.^16^ The use of simulation-based training in medical education has been extensively researched and proven to be effective across various clinical disciplines. It helped the students develop fast thinking and showed that learning had progressively improved with each session of simulation with a corresponding decrease in stress.^17–19^ Some studies have demonstrated that simulation-based training can improve ambulance nurses’ assessment and clinical management skills, especially in infrequent or high-risk clinical situations.^10,16^

In current ambulance nurse education, high-fidelity simulation is a frequently used method for students to convert theoretical skills into clinical practice. High-fidelity simulation is a didactic model that enables the representation of a real event and interactively evokes experiences of reality. The simulations sometimes include ethical issues. In nursing, various ethical problems can arise, for example, conflict problems where different ethical values are in conflict, attention problems, motivational problems, and structural problems, the problem lies in the structure rather than at the individual level.^20^

The learning preferably takes place in the reflection/debriefing after the scenario itself, which is part of the simulation, based on the diamond model.^21^ Souza and Vaswani^22^ suggest that a qualitative approach is preferred for the assessment which should take place through reflections, simulated patient interactions and the student’s individual knowledge development. Studies^23–25^ show that ethical simulation contributes to reflection on values, moral actions, and ethical values and contributes to increased knowledge both theoretically and practically. Simulation provides a chance to practice ethical decision-making, it is a safe learning environment where one can make mistakes and reflect on those mistakes without harming a real-life patient, it is a group activity that fosters collaboration. We developed a case scenario for the simulation that used a vulnerable population. We selected a vulnerable population because many ethical dilemmas can arise when providing care for these groups, and then perhaps list what those potential dilemmas may be. The students gain increased knowledge and a sense of being able to handle difficult ethical situations. Ethical simulation within specialist nurse education has been suggested as an innovative approach to enhance ethical decision-making skills and the development of a caring attitude in healthcare professionals, RN education, and SN education.^26^

### A model for care ethics

The model has been developed in response to the needs of nursing education and is based on several years of experience in teaching ethics within nursing education.^20^ The model is pluralistic in terms of underlying normative rationales. It has a consequentialist core, where the goal of nursing care should be the overall guide for decision-making and actions. However, these goals can only be arrived at considering a number of side-constraints, both more deontological constraints, as well as considerations of virtue ethics and caring ethics. By explicitly identifying and specifying the goal of care and a number of side-constraints to consider, the ethical competence of the nurse should be supported. However, these considerations cannot be automatically implemented without the use of the ethical competence of the nurse given the understanding that this competence is not exercised in a vacuum but influenced by a number of structural factors.

This goal, in terms of promoting patient health and quality of life, needs to be arrived at within several ethical side-constraints. Even if the patient is normally the priority, the interests and preferences of significant others need to be considered. Patient autonomy and participation are central in providing ethically acceptable nursing care. To be able to exercise autonomy, the patient needs autonomous ability in terms of authentic preferences and values, decision competence and some degree of action capacity (at least in terms of being able to communicate decisions^27^). Children are expected to develop this ability over time, and normally, adolescents can have a sufficient degree of such ability to make informed decisions. For smaller children, the legal guardians are expected to decide in line with what is in the children’s best interest and not primarily see to their own best interest. Care will involve an intrusion into the patient’s (and significant others’) privacy spheres, involving both the physical body, the physical space, and information about the patient and significant other. In trying to achieve the goal of care, patient identity, and understanding who the patient as a person is, are central. Given resources are scarce, resources need to be distributed in a fair and equal way, constraining what can be done to each individual patient. Overall, this needs to be done within the constraints of nurses, acting in a professional role.

**Figure 1.**
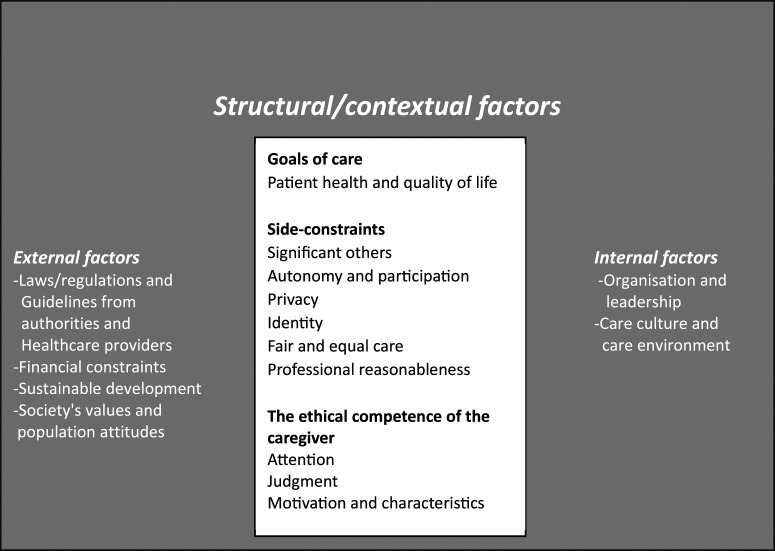
Schematic overview of the care ethical model adapted and translated from Etikboken, Sandman & Kjellström, 2024.

These explicitly ethical or normative considerations must be made within a structure or context, indirectly or directly influencing what will be possible or feasible but also actually done (at least when acting in an unreflected way). These are called structural or contextual factors and are both internal to the actual care situation in terms of organization and leadership for the actual care unit and the care culture of the unit, but also external in relation to the care unit. Such external factors are laws, regulations and guidelines the carers will have to relate to and abide by, financial constraints, aspects of sustainable development but also, in the end, societal values and population attitudes (Figure 1).

## Research aim

The research aim was to investigate the usefulness of high-fidelity simulation in ambulance specialist nurse education to teach ethical reasoning when caring for children.

## Research design

This study was conducted as a qualitative interview study, complemented with observations for data collection. Data was analysed using deductive qualitative content analysis according to Elo and Kyngäs.^28^ The study was conducted and reported following the consolidated criteria for reporting qualitative research (COREQ)^29^(Appendix 1). The reported settings and scenarios were used as a basis for data collection in a larger research project and the collected data were used in other research articles.

### Participants and research context

Participants were recruited from the ambulance nurse educational program at the University of Borås, Sweden. All students enrolled in the program during the fall term of 2022 were eligible to participate. The ambulance nurse students were registered nurses with a bachelor’s degree in caring science. Before the simulation studied here, they had completed two courses in the ambulance nurse educational program, including three to five simulation sessions based on the Advanced Medical Life Support (AMLS)^30^ concept. They also had approximately 2 weeks of clinical training in the ambulance service. Thus, the students were familiar with the simulated environment and the assessment structure. The ambulance nurse student’s clinical work experience varied, some had no prior experience in ambulance service or emergency healthcare, while others had several years of experience. A total of 35 participants (*n* = 35) were enrolled in the study with a mean age of 33 years (R = 25–43), 65% women (*n* = 23) and 35% men (*n* = 12). To obtain the opportunity to include the students in the study, both group interviews were carried out, as well as individual interviews a day or two later.

### Settings

The simulation sessions featured a scenario involving a child, 1.5-year-old, in need of emergency care, with two different versions of the scenario. The first scenario takes place in a well-kept apartment. Only the mother and child are at home. The mother becomes frightened and hysterical when the child suffers from febrile convulsions, but she participates as much as she receives in the care. The second scenario are localized to a blacked-out, messy apartment with both alcohol and drugs visible. The mother has another three-month-old child, who is doing well, and the 1.5-year-old child, who is unconscious due to febrile convulsions. The mother is under the influence of alcohol and does not want to cooperate with the ambulance staff. During the simulation, students either took on active roles as ambulance clinicians or served as observers, alternating these roles between the two scenario variations. For more detailed information regarding the simulation scenarios and equipment used, please refer to Appendix 2.

### Ethical considerations

The study has been reviewed and approved by the ethical council at the University of Borås (ref. no: FO2022/183). Furthermore, the Helsinki Declaration’s (World Medical Association, 2013) advice on ethical principles is followed. The participants received information about the study both verbally and in writing and they had the opportunity to ask questions and were informed that they could choose to cancel their participation at any time without further explanation. The facilitators who carried out field notes, group interviews and individual interviews had no connection to the teaching, implying that the participants were not dependent on the researchers who collected the data to pass the course.

### Data collection

#### Participant observation and field notes

During the simulation-based education day, one researcher collected data by documenting necessary contextual information in the form of field notes (*n* = 12).^31^ These notes were written during the scenario (i.e. what happened, what was said and by whom, body language, and placement in the room during specific events) and during the debriefing session (i.e. what was said, by whom, and visible expressions such as body language) after the simulation.

#### Group interviews

In addition to the observations and field notes, the same researcher conducted group interviews (*n* = 5) with selected participants (*n* = 16) following their simulation, with 2–4 participants in each group. The interviews lasted an average of 38 min (26–56 min) and were recorded and transcribed verbatim. All interviews followed a semi-structured interview guide (Appendix 3), posing the same initial questions but incorporating unique follow-up questions based on previous answers or discussions.

#### Individual interviews

Eight individual interviews (*n* = 8) were conducted with participants from five of the simulation groups within a week after the completed simulation session. These interviews were conducted by one author using a web-based communication application (Zoom). They followed the same semi-structured interview guide as the group interviews, including open-ended and follow-up questions. Each interview lasted an average of 17 min (12–25 min). The interviews were audio-recorded and transcribed verbatim for analysis.

### Analysis

Data was analysed using deductive qualitative content analysis^32^ where a care ethical model^20^ was used in creating the deductive categories accordingly. Data analysis was initiated after the transcription of interviews and used field notes as well as transcribed group and individual interviews as data. Initially, data was read through to get a sense of the whole. Meaning units were identified, according to the study aim and then condensed and gathered in codes. A structured categorization matrix based on the care ethical model (Figure 1) was developed to allocate the codes to each appropriate category. Within each category, data was abstracted and interpreted to form the results.

## Results

The results are based on eight individual interviews, five group interviews with two to four participants, and six sets of field notes, involving a total of 35 students in the specialized nurse education program with a focus on ambulance care. The results are presented categorically following the care ethical model deduction (Figure 1).

### Structural/contextual factors

#### Goals of care

The goals of care appear to be influenced by the design of the scenario. At the beginning of the scenario, the ambulance nurse students assess the care environment and identify problems that guide their initial actions. The ambulance nurse students set short-term goals for care related to the health of the sick child, although they are not always explicitly stated in the scenario itself but become apparent in reflection afterward. At the same time, some ambulance nurse students do not describe any goals for care at all, even during reflection. On rare occasions, there is a dialogue with colleagues or family members about the goals of care during the scenario and examples of disparate views of goals of care between ambulance nurse students and the mother were displayed. When the ambulance nurse students feel they have control over the child’s initial needs, some shift their focus and start considering more long-term goals for care in the situation. Some ambulance nurse students only mention long-term goals in the reflection after the scenario, while others do not reflect on long-term goals for care at all. As a secondary goal of care, some ambulance nurse students express a desire to instil trust and convey a sense of security to create conditions for good care. The long-term goals relate to the well-being of the entire family, not just the health of the sick child.

*“…the child who is unconscious has the greatest need, but the mother also needs help, and maybe the little one too?” *(#G2)

#### Side-constraints

The side-constraints that seem to have the most impact on the ambulance nurse students in the scenario are *significant others*, *autonomy* and *participation*. The child’s autonomy is seen as problematic in the current scenario since she is unconscious and unable to communicate her wishes (1.5 year old), and therefore, she is not considered decision-competent and autonomous. As a result, discussions about autonomy and participation are redirected towards the mother instead, as it is deemed important to involve her in the care and take actions to make her calm and secure in the situation. Since the patient is an incapacitated child, the involvement of relatives is seen either as an obstacle or an opportunity to manage the situation. There is variation in how the ambulance nurse students act: some communicate and include the mother in the decision-making process, while others choose to actively ignore the mother and solely focus on the child’s care, believing that the child has a right to care regardless of the mother’s opinion. When the mother does not exhibit what the ambulance nurse students perceive as good parenting, it can lead to anger and frustration, resulting in distancing themselves from the mother or displaying authoritarian behaviour towards her. Additionally, the ambulance nurse students’ previous experiences with similar situations as well as their prejudices can influence their perception of the importance of the mother’s participation, which is evident in their reflections after completing the scenario.

*“I didn’t think she should be so involved in the actual treatment in the acute situation. But… She can be involved in the situation and values, and that’s what I thought we achieved by having her observed and talking to her all the time.”* (#I1)

*“Yeah, it was the same for me, that thing with the mother, that she wasn’t present. I got the feeling that she didn’t care and how to handle that. That… It’s easier to push away that mother than involve her, as we did in the first case. To exclude her even more somehow.”* (#G1)

The ambulance nurse students do not reason about the child’s personal sphere and values, that is, the child’s *privacy* and *identity*. It can be described that the ambulance nurse students shape the care based on an assumption of what is best for the child. They consider that it is a child who should receive *fair and equal care* in accordance with the principle of human dignity, but they do not engage in reasoning based on principles of cost-effectiveness or principles of needs-solidarity. In their reflections about *professional reasonableness*, the ambulance nurse student’s express discomfort with the professional role and are unsure if the decisions they make are correct especially when they are forced to make decisions regarding the ethical dilemmas present in the scenario, taking over responsibility from the mother and not involving her in the decision-making. This discomfort and uncertainty in decision-making lead to pronounced ethical stress among the ambulance nurse students.

*“...it feels reasonable, I think, but of course, if the parents oppose it… Then it is. …It’s really difficult; you have to be absolutely sure that you have the legal support and that both parties in the crew agree on how the situation should be handled and all that.”* (#I4)

#### The ethical competence of the caregiver

The *ethical sensitivity/awareness* varies among the ambulance nurse students. Several of them identify the situation as ethically sensitive, while others do not see it as ethically problematic at all. Their own emotions often seem to dominate their judgement in decision-making, rather than ethical reasoning. The ambulance nurse students do not let ethical considerations dictate the care provided; instead, the physical needs of the child take priority. Variations in the ability to reason and make relevant decisions and actions occur. Different cues and information are considered relevant before decision-making. The variation may involve considering the complicated family situation, such as leaving a three-month-old baby at home with a drug-addicted mother. Additionally, the ambulance nurse students’ experience, knowledge, morality, and emotions play a significant role in decision-making and actions.

Expressions of the ambulance nurse students’ *ethical motivation* and *character* vary. Those who demonstrate reflection on ethical reasoning primarily describe the degree of quality of life as a decisive motivator for their actions, while others believe it is not their place to judge. Some argue that, as caregivers, they have the authority to have an opinion that the family in the scenario has no quality of life.

*“In my judgmental eyes, this is a poor environment to create a good quality of life. There is a greater likelihood that they will not thrive in this environment compared to others. That’s how I think.”* (#G1)

*“Well, quality of life can be so different. Some people experience quality of life by smoking a cigarette and eating chocolate. That might be the best thing they know, but… From a health perspective for children to grow up and have a good upbringing and be healthy and all - then it’s not optimal… so quality of life is not…” *(#G5)

*“The same goes there, the children don’t have a good life with their mother. I don’t think she leads a good life, but maybe she thinks her… experience is that she has a good life. //As for the children, we don’t really know. They might never have experienced anything else, so they might think this is a great life. But those of us who see things differently probably think they could have a significantly better life than what they have now.” *(#G1)

#### Internal structural factors

Regarding *organization* and *leadership*, support from other collaborative partners was considered to facilitate decision-making and actions, such as the police and social services in the current scenario. Since each partner are separate authority, they can provide support in various ways. The colleague within the ambulance team is considered the most important support in decision-making. The need for support, as an internal factor, appears to be experience-based – the less experience, the more support is requested.

During the decision-making process, one’s own actions and behaviour seem to be influenced by different norms and values, both the prevailing *care culture* within the healthcare team and the overall social culture within the ambulance organization. The ambulance nurse students claim that the prevailing norms are deeply ingrained and difficult to change. For example, how vigilant the caregivers are in noticing signs of neglect/abuse. The ambulance nurse students also state that signs of substance abuse issues in the *care environment* can create suspicion, trigger prejudice, affect ethical reasoning, decision-making, and consequently, the provision of care. Furthermore, it is emphasized that knowing the norms and values of one’s colleagues can be an asset.

*“…you talk a lot both in a regular workplace and in the ambulance, you get to know each other’s values and what baggage everyone carries… And that you don’t always have to tell or say much to each other, but you understand each other.”* (#I3)

#### External structural factors

The ambulance nurse students are fully aware that there are external structural factors that influence their decisions and actions. They primarily reason about *laws*, *regulations,* and *guidelines* during the scenario. Many are eager to act correctly but lack full knowledge of guidelines and the collaborating organizations and authorities, which contributes to uncertainty in decision-making and actions.

*“But regarding laws and such things. And then I think it becomes a bit difficult and a dilemma because I don’t know about all others, but at least I don’t have the entire law book in my head. I don’t know what applies in many cases. I would have difficulty, for example, figuring out whether we are legally allowed to take the child away from the mother because we think it is sick enough, or would we be committing an offense?”* (#G1)

The ambulance nurse students’ perception of *society’s values* and *attitudes from the population* is described as varying. Their understanding of what constitutes a correct way of living is based on the ambulance nurse students’ personal opinions about what is considered society’s norms. For example, situations involving substance abuse are seen to morally complicate ethical decision-making. It is described that caregivers may violate or overstep boundaries based on their own understanding of society’s norms and values regarding how caregivers should behave.

*“…in this situation, the violation is something that we are expected to do as licensed personnel.”* (#G2)

External structural factors that were not discussed or reasoned about include 1) supervisory authorities, county councils and municipalities, 2) financial constraints, and 3) sustainable development.

## Discussion

The usefulness of the simulation to stimulate the ambulance nurse students’ ethical reflection has, based on the ethical model analysis, proven to be feasible, as most of the students conducted well-founded ethical reasoning and reflected on ethics in an ambulance care context. Anderson et al.^33^ suggested that one way to support ethical reasoning in nursing education is simulation-based education, which can help to shed light on ethical problems and dilemmas and help to understand personal values and attitudes. According to Erbay,^34^ ambulance nurses are expected to have high ethical competence and make well-founded decisions in ethically complicated situations. Meanwhile, Haahr et al.^35^ believe that all RNs are challenged by organizational structures and the development of health care, which inhibits nurses’ professional decision-making and forces them to compromise on basic nursing values, such as ethical values.

At the time of the simulation, the ambulance nurse students typically have not gained in-depth knowledge of ethics and ethical reasoning, but the learning activity represents the introduction to training in theoretical and practical knowledge of professional ethics. The simulated session has a great impact on how students perceive the possibility of achieving the learning outcomes. If the session is perceived as unrealistic, students can experience difficulties in connecting concepts to action. However, even if they already possess professional ethical competence at a basic level as RNs it is obvious that this competence is varying. Juujärvi^36^ argues that ethical competence normally develops with training and education, but that different students still end up with different levels of competence, something which is needed to take into consideration in further training. One aspect of this, seen in our result, is the risk of applying one’s own views and values on the situation, and thereby risking to act paternalistic,^37^ instead of trying to take the perspective of those involved in the situation. Erbay^34^ claims that the ambulance care context is considered more challenging for the ambulance nurses than the intra-hospital care, as each situation is unique, and it is important that ambulance nurses identify ethical conflicts and at the same time provide appropriate care.

During the simulation sessions, the ambulance nurse students swiftly identified the child’s health problems and set short-term goals focussing on the care of the child. When they gained control over the situation some ambulance nurse students also considered more long-term goals of care, including trying to satisfy the health of the family as a unit, instilling trust, and creating a safe environment to achieve appropriate care. This might be related to development of ethical sensitivity,^1^ that is, being able to identify the relevant values at stake in the situation. Only focussing on the child’s health issues might be viewed as a limitation in sensitivity. However, starting with identifying the most acute needs in the situation and then widening the perspective, might be viewed as a more mature sensitivity, being able to prioritize what needs attention. The sensitivity in turn will impact on the reasoning and judgement the students will engage in and thereby also the actions. The students sometimes expressed that their goals with care varied, and they were not as keen to implement long-term goals when the parent showed signs of social despair such as problems with alcohol or drugs.

Another interesting finding is how the ambulance nurse students relate to autonomy in the situation. The child obviously lacks autonomous ability and hence, cannot exercise autonomy and thereby not have its autonomy respected. Hence, someone will have to act as a representative of the child.^38^ Normally, this is the parent. However, if the nurse judge that the parent does not take this responsibility, or act in their own interested rather than caring for the child, they might be ethically motivated to take over. Still, there is a risk that their own prejudice affects what they view as the child’s best interest and hence when the view the parent fails in this respect.^37^

Stigmatization of the mother could be an effect of the ambulance nurse student’s preconceived notions which seemed to influence their decision-making more than the desire to follow the to-do-good principle. Abelsson and Lindwall^39^ describe that ethical dilemmas affect ambulance nurses negatively because their own morals and attitudes are reflected in their actions towards the patient. Holmberg et al.^40^ argue that if ambulance nurses do not use their gut feeling as a professional ethical competence, it can prevent them from reacting to a situation that is unethical. Furthermore, ambulance nurses often lack the ability to ethically reflect on both the advantages and disadvantages of paternalism or stigmatization, which affects their ethical decision-making. Erbay^34^ showed that stigma affects patients with disabilities even before the first contact is made and can affect both the care given and caring actions, as it affects the ability to act because ambulance nurses are faced with an ethical conflict. In the current simulation sessions, the conflict consists of helping the sick child and at the same time trying to create long-term goals including an alcoholic mother who does not show any interest in participation. Further, Abelsson and Lindwall^39^ conclude that ambulance nurses who have learned to respect the patient’s dignity find themselves in a conflict of values, a personal inner conflict – a conflict they carry with them for a long time. In the simulation sessions, some students were affected emotionally, which can be explained by the fact that the ignorance of the mother leads to her degraded dignity. Sometimes healthcare professionals abandon the patient and do not care about the suffering person. They do not respect the patient and may even ignore the patient.^39^ As discussed above this could be interpreted as that the student’s prior knowledge, prejudice, attitudes, and views as well as confirmation bias affect their ethical reasoning and actions. By the design of the simulation, this was exposed during the sessions. The ambulance nurse students specifically expressed an increased awareness of their personal prerequisites during the debriefing sessions following the simulation.

The simulation sessions with associated debriefing stimulated most ambulance nurse students to an ethical reflection. They were forced to think about ethical concepts, such as autonomy and patient participation in a caring context, and to put words to ethical dilemmas and conduct and express ethical reasoning. However, there were ambulance nurse students who did not reflect at all and did not identify any ethical dilemmas during the simulation sessions. In a study by Abelsson and Lindwall,^39^ it became clear for the ambulance nurses when caring for a vulnerable patient, reality was completely different from their expectations. It takes courage and willpower to see the patient’s vulnerability without humiliating the patient. The ambulance nurses chose to do what was best for the patient without making it worse.^39^ Lack of courage and willpower can explain why some students do not choose to reflect on the ethical dilemmas of the simulation sessions. It could also be explained as a protective mechanism, such as the student preferring to shield himself from the problem and not see it, rather than dare to deal with it. Therefore, the session facilitator needs to pay attention to whether a reflection really takes place and preferably use an experiential reflection model to stimulate an ethical reflection. A conclusion that can still be drawn is that simulation contributes to ethical reflections and affects students’ learning positively.

## Limitations

Study credibility may be affected negatively during data collection since not all ambulance nurse students experienced the studied simulation at the same time. This could render a situation where the ambulance nurse students have a different number of simulation opportunities behind them and they can also exchange experiences with each other, which in turn can mean that some ambulance nurse students are more prepared for what awaits them during the simulation. What strengthens the credibility of the study is that individual interviews, group interviews and field notes were used, like a mixed method. The facilitators who performed the interviews and fieldnotes were not involved in the ambulance nurse education program, which means that the ambulance nurse students were not in a dependent position. Further, all researchers have contextual experience from ambulance care, higher education and simulation, and most of the facilitators also have experience in ethical simulation Reliability and accuracy were achieved by triangulating data^41^ from different sources among the authors, as all had access to the data.

## Conclusions

This study aimed to investigate whether high-fidelity simulations could be useful to stimulate ethical reflections among ambulance nurse students. The results showed that most of the students expressed some form of ethical reasoning during the simulation sessions, which were elaborated and reflected upon during the debriefing part of the sessions. The simulation design seemed to have a great impact on the outcome of the student’s ethical reasoning, where increased immersion led to deeper emotional engagement among the students which increased awareness of their personal preconceptions. In conclusion, a well-designed high-fidelity simulation can be useful as an educational tool to learn and enhance ethical competence among specialist ambulance nursing students. The knowledge gained from this study is likely to be transferable to other educational settings focused on developing ethical competence. The study’s findings could benefit educational providers in any nursing education setting who are looking for effective learning activities to teach ethical competence.

## Supplemental Material

Supplemental Material - Stimulating ambulance specialist nurse student’s ethical reflections by high-fidelity simulation Supplemental Material for Stimulating ambulance specialist nurse student’s ethical reflections by high-fidelity simulation by J Wihlborg, U Andersson, A Sterner, L Sandman, A Kängström and G Norberg Boysen in Nursing Ethics

Supplemental Material - Stimulating ambulance specialist nurse student’s ethical reflections by high-fidelity simulation Supplemental Material for Stimulating ambulance specialist nurse student’s ethical reflections by high-fidelity simulation by J Wihlborg, U Andersson, A Sterner, L Sandman, A Kängström and G Norberg Boysen in Nursing Ethics

Supplemental Material - Stimulating ambulance specialist nurse student’s ethical reflections by high-fidelity simulation Supplemental Material for Stimulating ambulance specialist nurse student’s ethical reflections by high-fidelity simulation by J Wihlborg, U Andersson, A Sterner, L Sandman, A Kängström and G Norberg Boysen in Nursing Ethics

## Data Availability

The datasets used and/or analysed during the current study are available from the corresponding author on reasonable request.*
