# Supplementary material for: Stimulating ambulance specialist nurse students’ ethical reflections by high-fidelity simulation
Source: Nurs Ethics. 2024 Oct 15;32(4):1197–209. doi: 10.1177/09697330241291162 (PMC12171055; doi:10.1177/09697330241291162)
Supplement: Supplemental Material - Stimulating ambulance specialist nurse student’s ethical reflections by high-fidelity simulation [file sj-pdf-2-nej-10.1177_09697330241291162.pdf]

Appendix 1

Description of SBE prerequsites. Simulation settings, scenarios and equipment.

The following text is based on the reporting guidelines for health care simulation research[1]. The reported scenarios were used as a basis for data collection in a larger research context, which were used in other research articles.

Participant orientation-orientation to the simulator, environment, location
Describe how participants were oriented to the simulator (e.g., method, content, duration). Orientation to the environment Describe how participants were oriented to the environment (e.g., method, content, duration). Describe where the simulation was conducted (e.g., in situ clinical environment, simulation center, etc.).

PREBRIEFING

The simulation took place in the University's simulation and training centre. The students began their orientation to the facilities, equipment and simulation as a pedagogical method during their introduction to the specialist nursing program. Which occurred approximately 11 weeks prior to the simulation. Furthermore, the students have also attended an orientation day at the simulation and training centre, where they received a guided introduction (approx. 2 hours) to the facility, equipment (i.e. emergency bags and stretchers) and the simulators with their functions and limitations. At this guided tour, the students are allowed to try out the equipment and the simulators for themselves.

A few weeks prior to the simulation the students are presented with written information regarding the specific condition of the patient in the simulated scenario (convulsion due to fever, paediatric patient). This allows them to prepare by reading the course literature. In addition, the students also receive and have access to the specific learning objectives and their related goals of the specific course. The information is also provided verbally and the students are allowed to ask questions during these information events.

About five weeks prior to attending the simulation the students have already performed 3 to 5 simulated scenarios within the scope of the program through an earlier course. These simulations were conducted in the same environment, with the same equipment and simulators, but the condition of the patients had a different focus. In summary, with prebriefing, briefing, scenario and debriefing the students had approximately 9-15 hours experience of simulation-based education.

Simulator make and model, simulator functionality
Describe the simulator make and model.
Simulator functionality Describe functionality and/or technical specifications that are relevant to the research question. Describe modifications, if any. Describe limitations of the simulator.

The simulator mannikin utilized is a Guamard Medical Pediatric HAL S3004. The simulator simulates a child of approximately 1-3 years of age and is dressed with a pyjama. The simulator has functions which are controlled by an operator, but they can also be scripted to change on specific timings. The functions are as follows; 1) breathing (frequency, depth, regularity, various sounds related to breathing and lungs), 2) swelling of the tounge, 3) varying degree of cyanosis around the mouth, 4) pulsations in brachialis and carotis artery (frequency, regularity, thickness), 5) auscultation of blood

[1] Cheng A, Kessler D, Mackinnon R, et al. Reporting guidelines for health care simulation research: extensions to the CONSORT and STROBE statements. Adv Simul. 2016;1(1):1-13

pressure (systolic and diastolic) in one arm, 6) eye functions such as blinking, pupil variation (dilated, small, fixed or light sensitivity), 7) convulsions and 8) speech through pre-recorded lines/sound or direct speech through operator microphone.
The following interventions can be done to the simulator; 1) create an unobstructed airway through chin thrust, nasal-cannula, pharyngeal tube, or laryngotracheal mask or intubation. 2) Auscultation of lung sounds and control of chest movement. 3) Peripheral venous catheter for i.v fluids and pharmaceuticals.
Limitations of the simulator are; 1) its rigidity, 2) latent transferring of audio from operator to simulator, 3) Skin, temperature, paleness/flushing, moisture, rashes and other markings on skin are not possible to produce on the simulator, 4) Capillary refill not possible, 5) not possible to create a bleeding and, 6) urine and faeces must be made up.

Simulation environment-equipment
Describe where the simulation was conducted (e.g., in situ clinical environment, simulation center, etc.).
Equipment Describe the nature of the equipment available (e.g., type, amount, location, size, etc.).

The simulation takes place in a specific room at the clinical training facility at the university. The room, approximately 15 sqm, is designed and furnished to portray an apartment.
In the first scenario, the apartment is bright and portrays a homely environment. A baby cot is placed by one of the walls, an armchair in the corner, a rug on the floor and a small table with a bottle of gruel on it. There is a play stove and toys neatly placed into plastic containers in the room. The baby cot is set with linen and a pillow.
In the second scenario, the apartment is furnished the same, but nothing is neatly put away and the lights are off or very dimed. In addition, there is visible cigarettes, ashtray, beer cans, a wine bottle, liqueur bottle and a used cannula.
During the simulation the students utilizes two emergency bag in which the equipment is adapted for assessing and treating paediatric patients in the prehospital context. Available equipment includes; various pharmaceuticals, syringes and cannulas for various administration lines (peripheral or intraosseous), thermometer, blood pressure cuff, pulse oximeter, measuring blood sugar. There is also equipment for securing and managing an airway, treatment guidelines in A5-format and a short version of a standardized assessment according to the AMLS-structure. In addition, there is also an ambulance stretcher. The equipment is designed for singular and sterile use but is reused to save resources, reduce waste and preserve the environment.

Simulation event-scenario
List the learning objectives and describe how they were incorporated into the event.
Describe if the event was programmed and/or scripted (e.g., orientation to event, scenario progression, triggers). If a scenario was used, the scenario script should be provided as an appendix.

The simulation is built upon a simulation guide which are utilized at the University's clinical training centre (See appendix)
The specific goals for the scenario are:

- Students should conduct a primary assessment based on the S A-E (AMLS)
- Students must discover and remedy life-threatening conditions if needed.
- Students must produce a focused anamnesis from close ones, if present
- Student must conduct a focused assessment based on what they perceive as important for the scenario.
- Students must measure patient vital signs considered important.
- Students must treat the patient based on what is demanded in the situation but also in relation to available resources and time.
- Students must, if considered possible, begin transportation (simulated) to an emergency department

Appendix 1

- Students must conduct a handover report to charge nurse at the receiving unit. The report should contain current status, underlying cause for symptoms and provided and ongoing treatment(s)

BRIEFING
As the scenario starts the students are introduced while standing in a corridor outside of the specific room (apartment). They receive an ambulance call to a fictitious address with information that the call regards a 1,5-year-old girl who is unconscious and possibly having convulsions. The mother is at the scene. The time of day is 07:50 and it's a Tuesday morning. After receiving this information, the students are given a few minutes to ´drive to the address´ and enable some discussion for possible preparations. Total time for briefing is approximately 10 minutes.

Group vs individual practice-use of adjuncts, media.
Describe if the simulation was conducted in groups or as individuals.

In each simulation session there is a group of 3-4 students. Two of the students attend the simulation bedside and 1-2 students are observers. The observers are located together with a facilitator in an adjacent room to which the scenario is streamed to a projector screen along with audio. The facilitator also operates the simulator. At the end of the first scenario session, the students switch roles so that each student have taken part in at least one of the two scenario versions. Both scenarios include briefing, scenario, and debriefing.

Facilitator/operator characteristics
Describe experience (e.g., clinical, educational), training (e.g., fellowship, courses), profession.

The facilitators are employed at the institution for caring science since several years (8-11 years). They are registered nurses with a specialist education towards prehospital emergency care, and have approximately 20 years of experience from the field. In terms of experience from conducting simulation-based education they both have approximately 6 years' experience. They have both finished an instructor course in medical simulation (IMS) and an additional local course for operation of the specific simulator utilized.

Pilot testing
The current scenario has been used in the specialist education towards prehospital emergency care for three consecutive years. The scenario is built to assist reflection during debriefing and relates to the vulnerable patients. There has been continuously assessment of the scenario for developmental purposes.

Actors/confederates/standardized/simulated patients
Describe experience (e.g., clinical, educational), training (e.g., fellowship, courses), profession, sex. Describe various roles, including training, scripting, orientation, and compliance with roles.

Please see the description of the facilitators and operators for information. There was no formal script for the scenarios beside the initial simulation protocol with the vital signs. The facilitators were free to act upon the interaction of the students and their prior experience of clinical work and knowledge.

Instructional design (for educational interventions) or exposure (for simulation as investigative methodology)
Duration, describe the duration of the educational intervention. If the intervention involves more than one segment, describe the duration of the segment.

Th simulation session takes about 3 hours, which includes briefing (10 minutes), scenario (20-25 minutes) and debriefing (50 minutes).

Appendix 1

Timing

Describe the timing of the educational intervention relative to the time when assessment/data collection occurs (e.g., just-in-time training).

The simulation was conducted during the students third course in the specialist nursing program. The data collection occurred during the debriefing session and in study specific group or individual interviews. Group interviews was conducted directly after debriefing, and individual interviews were conducted 3-10 days after the simulation session.

Frequency/repetitions

Describe how many repetitions were permitted and/or the frequency of training (e.g., deliberate practice).

The students had not taken part in the specific scenario(s) prior to their simulation. Instead, they had 3-5 simulation sessions in the prior courses of the educational program.

Clinical variation, Range of difficulty

Describe the variation in clinical context (e.g., multiple different patient scenarios). Describe the variation in difficulty or complexity of the task.

The scenario tasks that the students are required to solve is complex and multifaceted. The must assess the situation and the patient and take necessary actions based on their findings. The two scenarios are similar but with some variance in relation to what is making it more complex.

In the first scenario, the student encounters a very worried mother who expresses this by screaming and crying while rapidly trying to hand over her child to the ambulance. The child is seemingly motionless and cyanotic. The students are required to being their assessment in order to locate and treat any potential life-threatening conditions. The child is breathing on its own, but are in need of oxygen. If the students take a focused anamnesis from the mother, they will learn that the child has a cold and that the mother recently found the child becoming rigid and then the face turning blueish only for the child to be motionless. During their assessment the students will find a heightened body temperature, which in turn would need medication. The environment surrounding the child and mother is clean, tidy and bright. The mother portrays a genuine and true worry regarding the health of the child. It is important to confirm the mother's feelings and provide support.

In the other scenario, the students must knock on the door to the apartment. The door only opens for a few centimetres, its gloomy and dark in the apartment. There is a mother holding a three-month-old infant in her arms, she immediately asks the question about who they are and who have been calling them. The mother unwillingly lets the students inside the untidy and dark apartment. A bit further into the apartment another child (the patient) is lying on the floor, motionless and with cyanosis. The students must here immediately begin their primary assessment of the child in order to detect and treat life-threatening conditions. Besides the cyanosis, which requires oxygen treatment. There is also a bandage on the back of the child's head. The mother, who lives alone, have an untidy apartment with visible beer cans, wine bottles, a box with narcotic pharmaceuticals are located on tables. In this scenario the mother is not worried at all for the child on the floor, but is rather more concerned that the three-month-old baby is going to wake up. She unwillingly responds to questions asked by the students, and does not want to travel to the hospital as she is tiered from a rough and long night after attending the older child at a previous hospital visit. In this scenario, the students should start to reason regarding potential social neglect and that there might be need for support from social services.

Standards/assessment

Appendix 1

Describe predefined standards for participant performance (e.g., mastery learning) and how these standards were established)

The student must be able to begin a structured assessment based on the AMLS –concept. This requires a primary assessment to detect and treat life-threatening conditions. In addition, there needs to be a reflection on whether or not detected condition(s) are life-threatening, critical or non-critical. The students must also discuss potential differential diagnoses. The must move on to a secondary assessment which is more focused towards diagnosis and final treatment. In their assessment students must reflect upon differences between assessing and treating adults and children.
The debriefing poses an opportunity for learning through reflection upon one's own and others actions. In order to gain a pass on the simulation session, the students must actively take part in all parts of the simulation and the students are also assessed based on Paramedic Global Rating Scale[2]. If the students should not receive a pass at their first attempt, there is a possibility to retake the simulation session once more during the course.

Adaptability of intervention, integration
Describe how the training was responsive to individual learner needs (e.g., individualized learning). Describe how the intervention was integrated into curriculum.

After the course the students should be able to fulfil the following course goals:
- Apply theories and models related to identifying, observing, and assessing patients that suffers from acute illness or injuries. Also, evaluate patients that might be particularly vulnerable or exposed. Based on the condition the student must be able to initiate, conduct and evaluate measures taken and caring that is required by the patient condition.
- Reflect on dilemmas connected to the caring of patients suffering from sudden illness or injury and who are assessed as particularly vulnerable or exposed.

Feedback and/or debriefing
Source Describe the source of feedback (e.g., computer, simulator, facilitator). Duration Describe the amount of time spent. Facilitator presence Describe if a facilitator was present (yes/no), and if so, how many facilitators.

DEBRIEFING
After each scenario the students, observers and facilitators gather for debriefing. The debriefing is conducted according to the Diamond-model, focusing on the feeling residing in the students directly after finishing the simulation. The acting students gets to describe what they experienced happened in the scenario. The observing students are also provided space to share their observations. The discussion also touches on whether the students would have done anything different if they had done it all again. These reflections can be connected to the course literature, laws, competence guidelines. In addition, the facilitators have a few bullet-points that are discussed also; Why adults do not have fever convulsions and how these are treated among children.; There is also a discussion of what could be done if the parent would not have opened the door for the students; What could a police patrol assist with; When is a report to the social services obligated; In what ways and how does one's own preconceptions influence the encounter and how we act. Finally, all students must answer as to what they will take with them from the scenario into their future practice. The debriefing session lasts approximately 50 minutes.

[2] https://pubmed.ncbi.nlm.nih.gov/32768897/
