# Supplementary material for: Stimulating ambulance specialist nurse students’ ethical reflections by high-fidelity simulation
Source: Nurs Ethics. 2024 Oct 15;32(4):1197–209. doi: 10.1177/09697330241291162 (PMC12171055; doi:10.1177/09697330241291162)
Supplement: Supplemental Material - Stimulating ambulance specialist nurse student’s ethical reflections by high-fidelity simulation [file sj-pdf-3-nej-10.1177_09697330241291162.pdf]

Appendix 3

Interview guide for group/pair/individual interview:

a) Record gender:

b) Ask about age:

c) Experience as a registered nurse:

d) Experience in ambulance service:

e) Which case did you simulate: A (Gabbe) B (Anna)

f) Were you caregiver 1 or 2: 1 2

1. What did you feel during the simulation? Why do you think you felt that way?
2. Which decision(s) did you find the hardest to make in these scenarios? Why?
3. Did you have any goals for the care provided? If so, what were they?
   (Goals of care)
4. Did you perceive any ethical aspects to consider in the scenario? Which ones? Were any of these more important to consider?
   (Which ethical values are prioritized: quality of life and a good life)
5. How did you reason/think about whose needs you need to consider in the scenario? In what ways are these needs met? *(Relatives, autonomy/participation, integrity, identity, justice/equality, reasonableness in the work role)*
6. What knowledge would you say is needed to handle the scenarios in the current simulation?
   (Ethical competence)
7. Are there any environmental factors that affect your actions in the scenario?
   (Current organization and leadership, care environment/culture, laws and regulations, economic frameworks, sustainable development, societal views, etc.)
8. Has this simulation developed/affected your ethical thinking/reasoning? In what way?
9. What do you think this simulation has contributed to, for you?
10. If you could redo the simulation, is there anything you would do differently? If so, what?
11. How do you/your team feel the cooperation worked?
12. Did you/your team feel any fears/uncertainties before and during the scenario?
13. How realistic did you/your team find the scenario?
